# Computational model of a vector-mediated epidemic

**DOI:** 10.1119/1.4917164

**Published:** 2015-05

**Authors:** Adriana Gomes Dickman, Ronald Dickman

**Affiliations:** Programa de Pós-graduação em Ensino de Ciências e Matemática, Pontifícia Universidade Católica de Minas Gerais, Av. Dom José Gaspar, 500, Coração Eucarístico, 30535-901, Belo Horizonte, Minas Gerais, Brazil; Departamento de Física and National Institute of Science and Technology of Complex Systems, ICEx, Universidade Federal de Minas Gerais, Caixa Postal 702, 30161-970, Belo Horizonte - Minas Gerais, Brazil

## Abstract

We discuss a lattice model of vector-mediated transmission of a disease to illustrate how simulations can be applied in epidemiology. The population consists of two species, human hosts and vectors, which contract the disease from one another. Hosts are sedentary, while vectors (mosquitoes) diffuse in space. Examples of such diseases are malaria, dengue fever, and Pierce's disease in vineyards. The model exhibits a phase transition between an absorbing (infection free) phase and an active one as parameters such as infection rates and vector density are varied.

## I. INTRODUCTION

The spread of epidemics is an urgent problem in medicine and public health. The threat of an Ebola outbreak, the increasing number of people with AIDS, and the observation that diseases such as malaria and influenza still kill many people worldwide, justifies its importance. In epidemiology—the study of the occurrence, transmission, and control of disease—mathematical models are an important tool for quantifying spreading patterns and understanding the transmission process. In this paper, we discuss an epidemic model in which transmission is mediated by a vector, illustrating the application of ideas from statistical mechanics beyond the context of thermodynamics.

In many regions, epidemics of malaria, dengue fever or yellow fever are recurrent, costing many lives and resources in efforts to treat and possibly eradicate the disease. The spread of these diseases depends on a vector that transmits a parasite to humans, and in some cases, to animals. The vector is an infected female mosquito. The dynamics of the human-vector interaction can be summarized as follows. An infected vector, whose saliva contains the parasite, transmits the latter to a human host through a bite. Once in the host, the parasite passes through several stages, until it migrates to the red blood cells. A vector can become infected upon biting an infected host. Within the vector, the parasite also undergoes several phases and finally migrates to the salivary glands, repeating the cycle.

In some cases, the predictions of mathematical models can guide immunization programs, or influence the choice of techniques to eliminate the disease.^1^ The epidemiology of any disease is too complex to be described by a single model, which, according to Ref. 2, “should be used to identify and answer specific questions.”

The first model of transmission of a vector-borne disease was formulated by Ross in 1911.^3,4^ Ross considered the dynamics of two populations, human hosts and vectors, ruled by rates of recovery, birth, death and biting, and described by a pair of differential equations for the densities of susceptible and infected individuals. Ross identified the existence of a threshold vector density, below which the population would be free of the disease in the long-time limit. He concluded that programs to control the disease should concentrate on keeping the vector population below a limiting value.

In this paper, we introduce a lattice model for vector-mediated transmission of a disease in a population consisting of two species, human hosts and vectors (mosquitoes), which contract the disease from one another. Hosts are sedentary, while vectors diffuse in space. Our model is based on Ross' work but includes spatial structure and the diffusion of vectors. It is an agent-based stochastic process, in contrast to Ross' model which treats average densities in a deterministic fashion. Thus, our model includes fluctuations, which are ignored in Ross' mean-field approach. We investigate the dynamic behavior of the model using Monte Carlo simulations.

In the following, we discuss the construction of the model, basic critical behavior concepts and absorbing-state phase transitions, special features of the algorithm, and some of our results.

## II. MODELING A VECTOR-BORNE DISEASE

Models describing the transmission of a vector-borne disease are based on the parasite life cycle, alternating between the host and the vector. Hosts can be infected only through the bite of infectious vectors, and vectors can become infected only by biting infectious hosts. Hence, a model of a vector-borne disease must include host and vector populations. From now on, we refer to the human host simply as the host and to mosquitoes as vectors.

In simpler models, the sizes of both populations are fixed, comprising *N_h_* hosts and *N_v_* vectors. Because hosts typically live much longer than vectors, we interpret the invariability of the populations differently. For hosts, it means that birth and death are not important on the time scale of the epidemic. The fixed vector population size follows from the simplifying hypothesis that each vector that dies is immediately replaced by a new, uninfected one.

The arrival of disease-free vectors is represented by the vector replacement rate. We note that even if the average vector population were constant in the region of study, we would expect it to fluctuate about its mean value. In the model, fluctuations of the total number of vectors are ignored; locally, there are fluctuations as vectors hop between lattice sites.

The model is defined on a lattice of *N* sites. It is a stochastic model, more specifically, a Markov process, defined by its state space and a set of transition rates between different states. At each lattice site there is a host, which can be in one of two states, infected or healthy. (Thus *N_h_* = *N*.) Each site can either be free of vectors or occupied by infected and/or healthy vectors. Thus, the state or configuration of the model is specified by the following set of random variables. At each site *j*, we define *h_j_* = 0 or 1 representing, respectively, a healthy or infected host at that site, and nonnegative integers $v_{0,j}$ and $v_{1,j}$ representing the number of healthy and infected vectors, respectively. Because the total number of vectors is fixed, we have $\sum_{j=1}^{N}\left(v_{0,j}+v_{1,j}\right)=N_{v}$.

The dynamics of the model is given by the following rules:

• Vectors hop to a neighboring site at a rate *D*.
• A healthy vector becomes infected at rate *I_v_* if it shares a site with an infected host.
• An infected vector is replaced by a healthy one at rate *R_v_*.
• A healthy host at site *j* becomes infected at rate $v_{1,j}I_{h}$.
• An infected host recovers at rate *R_h_*.

These rules are readily translated into transition rates. Suppose, for example, that C is a configuration with vector numbers $v_{0,j}$ and $v_{1,j}$ at a site *j*, and C′ is the configuration with $v\prime_{0,j}=v_{0,j}-1$ and $v\prime_{1,j}=v_{1,j}+1$; all other variables are identical to those of C. The second rule implies the transition rate $W(C\to C\prime )=I_{v}h_{j}v_{0,j}$. The other dynamic rules can also be expressed in terms of transition rates. Note that the rate for a vector to hop from site *j* to a neighbor site *k* is $D/z_{j}$, with *z_j_* the number of sites that are neighbors of site *j*. Although we restrict our attention to regular lattices in the present work, the model can be implemented on any lattice or network structure; the network is specified by the list of pairs of neighbors.

Vector diffusion is the only mechanism for spreading the infection in the host population. Similarly, the parasite passes from one vector to another only via an intermediary host. We assume that any infected individual is also infectious, that is, an individual becomes infectious the moment it is infected. This simplification implies that we are not able to include information about incubation periods.

Our model is closely related to one defined by Macnadbay *et al*.^5^ These authors use computer simulations to study the critical behavior of an epidemic in which the vector population is allowed to diffuse on the lattice, infecting a static population upon contact. Thus, in their model, individuals become infected instantaneously. In our model, the infection rate is finite, so that a healthy host may share a site with infected vectors, and vice-versa.

## III. BASIC CONCEPTS

In this section, we introduce some of the concepts of nonequilibrium phase transitions. For this purpose, we introduce the contact process, the simplest spatial model exhibiting a phase transition between an active and an absorbing state.

In the contact process,^6–8^ a Markov process that can be interpreted as a model for the spread of an epidemic, each site of the lattice represents an individual that may be infected or healthy. The infection spreads by direct contact between infected and healthy individuals, that is, between sites that are neighbors on the lattice. Infected individuals recover at unit rate and are then susceptible to reinfection. A healthy individual at site *i* becomes infected at rate $n_{i}\lambda /z_{i}$, where *n_i_* is the number of infected neighbors. Because an individual must have at least one infected neighbor to become infected, the state in which all the individuals are healthy is *absorbing*; that is, the system cannot escape from this configuration.

Persistence of the epidemic is controlled by the infection parameter *λ*. If *λ* is small, extinction at long times is certain; large values of *λ* assure that the infection spreads indefinitely. The boundary between persistence and extinction is marked by a critical point, denoted as *λ_c_*. The critical parameter *λ_c_* separates the two possible steady states the system can reach asymptotically, namely a disease-free or absorbing state and a surviving epidemic or active state, in which the stationary density of infected individuals, *ρ*, is nonzero. It turns out that *λ_c_* marks a continuous phase transition. Because *ρ* is zero in one phase and is greater than zero in the other, it can be identified as the order parameter of the transition, just as the magnetization is the order parameter for a ferromagnetic-paramagnetic phase transition. At a continuous phase transition the order parameter increases continuously from zero as the infection parameter is increased beyond its critical value. As *λ* approaches its critical value from above, the order parameter approaches zero as a power law, $\rho ∼{\left(\lambda -\lambda_{c}\right)}^{\beta }$. The asymptotic scaling of *ρ* is characterized by the critical exponent, *β*. Near the critical point, the system exhibits strong fluctuations, correlated over large times and distances.^9,10^ The correlation length *ξ* diverges at criticality as

$$\xi ∼|\lambda -\lambda_{c}{|}^{-\nu_{⊥}},$$ (1)

where $\nu_{⊥}$ is the correlation-length exponent. The relaxation time, *τ*, the time it takes for a system to reach the steady state, diverges as

$$\tau \propto |\lambda -\lambda_{c}{|}^{-\nu_{\|}},$$ (2)

where $\nu_{\|}$ is the relaxation-time exponent.^11–13^

Another fundamental aspect of absorbing-state phase transitions is the propagation of activity in space and time, starting from a configuration having a single active site at the origin.^14^ (The spread of an epidemic starting from a single infected individual is of great interest in epidemiology.) Here, we focus on *P*(*t*), the probability that the system has not entered the absorbing state at time *t*, and *n*(*t*), the mean number of infected individuals. In the subcritical regime, $\lambda <\lambda_{c}$, *P*(*t*) and *n*(*t*) decay exponentially. In the supercritical regime, there is a nonzero probability that the activity persists indefinitely, so that $P(t)\to P_{\infty }>0$ as t→∞, and $n(t)\propto t^{d}$ in a *d*-dimensional system. At the critical point, the process dies out with probability one, but the mean lifetime diverges. In the absence of a characteristic time scale, the asymptotic evolution follows power laws:

$$P(t)\propto t^{-\delta },$$ (3)

$$n(t)\propto t^{\eta },$$ (4)

where *δ* and *η* are additional critical exponents. The power-law dependence of *P* and *n* on time provides an effective criterion for estimating *λ_c_* in numerical studies.

The independence of the critical exponents on most details of the system reflects universality in critical phenomena. Models with the same set of critical exponents form a universality class. In general, a universality class is determined by global features such as dimensionality, symmetry group of the order parameter, and the range (long or short) of the interactions. Models possessing a continuous transition to an absorbing state generally belong to the same universality class as directed percolation.^15–18^ The presence of a conserved quantity can alter critical behavior.^19,20^ Although there is no proof, we expect the vector-borne epidemic model to show qualitative behavior analogous to that of the contact process, because it also exhibits a phase transition between an active and an absorbing state. Because the total vector population is conserved, we might expect the model to exhibit non-directed percolation scaling. It remains an open question to which universality class the vector-borne epidemic model belongs.

## IV. SIMULATION ALGORITHMS

We now turn to the elaboration of algorithms for simulating the contact process and the vector-borne epidemic. Both models are Markov processes defined in continuous time (that is, in terms of transition rates). In continuous-time processes elementary events, such as infection and recovery in the contact process, occur one at a time; two such events never occur simultaneously.^21^

To construct the algorithm, we need to answer three questions. Given the current configuration of the system: (1) what kind of event will occur next?, (2) where will it occur?, and (3) when will it occur?

### A. Contact process

In the contact process each infected site has a rate of unity to recover and a rate of *λ* to attempt infecting a nearest-neighbor site. Thus if there are *N*_1_ infected sites, the total transition rate is $r=N_{1}(1+\lambda )$, which means that the time *s* to the next event is an exponentially distributed random variable, $p(s)=re^{-rs}$. In many cases, we simply replace the random variable *s* by its mean, 1/r, and advance the time counter by this amount at each step.^22^ To decide where the event will take place, we choose one of the *N*_1_ currently infected sites at random, say *j*, by randomly generating an integer uniformly distributed between 1 and *N*_1_. To choose the type of event, we note that a given event is recovery with probability $p_{d}=1/(1+\lambda )$ and is an infection attempt with the complementary probability, $1-p_{d}$. Thus, we generate a uniform random number *y* in the interval [0,1) and recover the chosen site *j* if $y<p_{d}$. If $y\geq p_{d}$, we choose one of the *z_j_* nearest neighbors of site *j* and infect this site if it is currently uninfected. (If the chosen neighbor is already infected, no changes are made to the configuration at this step.) Whenever the configuration changes, the list of infected sites must be updated. Despite a small overhead associated with maintaining the list, this algorithm provides a highly efficient method for simulating the contact process.

*Problem 1*. Explain why it would be incorrect to use the same time increment, independent of *N*_1_, at each step, in the algorithm we have described.

*Problem 2*. Explain why it would be incorrect to choose another nearest neighbor of site *j* if the first choice yields an already infected site.

*Problem 3*. Write a set of commands to remove an uninfected site from the list of infected sites; the number of operations should be independent of the list size.

### B. Vector-borne disease: Continuous-time algorithm

The algorithm used to simulate a vector-borne epidemic model is considerably more complicated than that used for the contact process, because we have two classes of individuals in the process. To choose the next event, we note that the total transition rate *R* is the sum of the transition rates for five possible events

$$R=R_{h}H_{1}+R_{v}V_{1}+DN_{v}+I_{v}\sum_{j}h_{j}v_{0,j}+I_{h}\sum_{j}\left(1-h_{j}\right)v_{1,j},$$ (5)

where the terms represent host recovery, vector replacement, vector hopping, vector infection, and host infection, respectively, *V*_1_ is the number of infected vectors and *H*_1_ the number of infected hosts. We refer to these as events of type 1, …, 5, respectively.

The probability that the next event is of type *k* is given by the ratio of its transition rate to the total rate, so that, for example, the probability that the next event is recovery of a host is

$$p_{1}=\frac{R_{h}H_{1}}{R}.$$ (6)

To choose the next event type, we generate a uniform random number *y* in the interval [0,1), as in the contact process algorithm. If $0\leq y<p_{1}$, the next event is of type 1; if $p_{1}<y<p_{1}+p_{2}$, it is of type 2, and so on.

To implement this scheme, we need lists of infected hosts, infected vectors, pairs of infected hosts and healthy vectors occupying the same site, and pairs of healthy hosts and infected vectors occupying the same site. The lists need to be updated following each event. Moreover, for diffusion, we require an array storing the current position of each vector.

The algorithm involves the following steps:

(1) Initialize the system, defining the states (infected or not) of each host and vector, and distribute the vectors over the lattice, randomly.
(2) Determine which kind of event will occur next.
(3) Choose the entity (for example, infected host) involved in the event from the appropriate list.
(4) Following the event, update the lists and (in case of diffusion) vector positions, and increment the time, t→t+1/R.
(5) Go to step 2.

### C. Vector-borne disease: Discrete-time algorithm

The algorithm we have described for a vector-borne disease involves considerable expenditure for choosing events and maintaining lists. We turn now to a simpler algorithm involving discrete time: in this case the entire system is updated simultaneously in a pass of small but finite duration, Δt. The algorithm employs the variables $v_{0,j}$ and $v_{1,j}$, and a logical variable *k_j_* which is true if site *j* harbors an infected host and is false if not. There is no need to record individual vector positions or maintain lists of the kind used in the continuous-time approach.

Once the initial states of the individuals have been defined and the vectors distributed over the lattice, the evolution proceeds in a series of substeps:

*Host recovery/infection and vector infection*. At each site *j*, if the host is infected, then the host recovers with probability $r_{h}\equiv 1-\text{exp}(-R_{h}\Delta t)$. If there are uninfected vectors at site *j* (that is, $v_{0,j}>0$), *n* of them become infected, where *n* is a binomial random number with

$$P(n)=\left(\begin{array}{c} v_{0,j}\\ n \end{array}\right){\left[1-\text{exp}(-I_{v}\Delta t)\right]}^{n}\;\text{exp}[-(v_{0,j}-n)I_{v}\Delta t]$$ (7)

for $n=0,1,2,\ldots ,v_{0,j}$.

If the host at site *j* is not infected, and the site harbors infected vectors, the host becomes infected with probability $1-\text{exp}(-v_{1,j}I_{h}\Delta t)$.

*Vector replacement*. At each site *j*, if there are infected vectors, *n* of them are replaced with uninfected ones, where *n* is a binomial random number with

$$P(n)=\left(\begin{array}{c} v_{1,j}\\ n \end{array}\right){\left[1-\text{exp}(-R_{v}\Delta t)\right]}^{n}\;\text{exp}[-(v_{1,j}-n)R_{v}\Delta t]$$ (8)

for $n=0,1,2,\ldots ,v_{1,j}$.

*Vector hopping*. At each site *j*, if there are infected vectors, *n* of them hop, where *n* is a binomial random number with

$$P(n)=\left(\begin{array}{c} v_{1,j}\\ n \end{array}\right){\left[1-\text{exp}(-D\Delta t)\right]}^{n}\;\text{exp}[-(v_{1,j}-n)D\Delta t]$$ (9)

for $n=0,1,2,\ldots ,v_{1,j}$. Choose the new site for each hopping vector from the set of neighbors of site *j*. Apply the same procedure to the uninfected vectors.

The binomial probability distributions associated with vector infection, replacement and hopping are stored in lookup tables. To avoid multiple hopping of the same vector in a single step, all hopping events are generated before any vectors are actually transferred. For each site, let $\Delta_{1,j}$ be the change in the number of infected vectors at site *j* due to hopping; at the beginning of the hopping substep, all the $\Delta_{1,j}$ are set to zero. Suppose, for example, that two infected vectors are to be transferred from site *j* to j′. Then we let $\Delta_{1,j}\to \Delta_{1,j}-2$ and $\Delta_{1,j\prime }\to \Delta_{1,j\prime }+2$. Once all sites have been visited in the hopping substep, we let $v_{1,j}\to v_{1,j}+\Delta_{1,j}$ for each site *j*. Naturally, the same procedure is applied to the uninfected vectors.

In this discrete-time simulation, many events can occur in a single sweep of the lattice. We nevertheless want to keep the time increment Δt sufficiently small such that the probability of recovery and subsequent reinfection of the same individual during the same step is small. Thus if $R_{\max}$ is the largest of the rates (of infection, recovery/replacement, or hopping), we need to have $R_{\max}\Delta t\ll 1$. For instance, in the studies discussed in the following $R_{\max}=I_{v}=2$ and Δt=0.1. The results of the discrete- and continuous-time simulations agree in the limit Δt→0, but letting Δt be very near zero is impractical: enormous cpu time would be spent while almost nothing happens. Because we must use a finite Δt, the value of a critical parameter such as *λ_c_* will be somewhat different in the discrete- and continuous-time simulations. However, the results for universal properties such as critical exponents should be the same for both methods.

Because the discrete-time scheme is somewhat complicated, we tested it first on the one-dimensional contact process. For Δt=0.1, we obtained $\lambda_{c}=2.81$, compared with the known value of $\lambda_{c}=3.297848$ for the continuous-time process.^8^ Despite this difference, the discrete-time simulations yield critical exponents that agree with the known values for the contact process.

The methods we have described produce a single realization of the process. A given realization ends when either the process has reached an absorbing configuration, or some maximum time $t_{\max}$, defined by the user, is attained. In spreading simulations, the process must be repeated a large number of times, $N_{\text{rep}}$, to obtain good statistics for *P*(*t*) and *n*(*t*). A typical strategy is to declare vectors *P*(*j*) and *n*(*j*) for each integer time *j* from zero up to $t_{\max}$. In a given realization, these variables are updated whenever the simulation time variable hits (or just surpasses) the next integer value *j*, so P(j)→P(j)+1, and $n(j)\to n(j)+n_{1}$, where *n*_1_ is the number of infected individuals. (In the vector-borne epidemic model, we require two counters, $n_{h}(j)$ and $n_{v}(j)$, for hosts and vectors, respectively.) Once all realizations are completed, normalizing these vectors by $N_{\text{rep}}$ yields the survival probability and mean infected population size.^23^

## V. RESULTS

In the following, we provide some illustrative results for the vector-borne epidemic model in one dimension; more definitive conclusions on critical behavior will be given elsewhere.^24^ (Although it might seem more realistic to use a two-dimensional lattice to map out a city or region in which the epidemic takes place, we can think of the one-dimensional case as representing a population living along a river.)

A fundamental piece of information about the process is the survival threshold; that is, the surface in parameter space separating the regime in which the epidemic persists from that in which it dies out. As discussed, this critical surface marks a continuous phase transition. Given the large number of parameters, we do not propose to delineate this surface completely. We shall instead fix most of the parameters, and select one as the control parameter and seek its critical value. By repeating this analysis using different parameter values, we can trace out the critical surface.

For both continuous and discrete-time simulations, we study spreading from a single infected individual. Thus, the initial configuration is characterized by one infected host. We adopt periodic boundary conditions for the lattice.

**Fig. 1.**
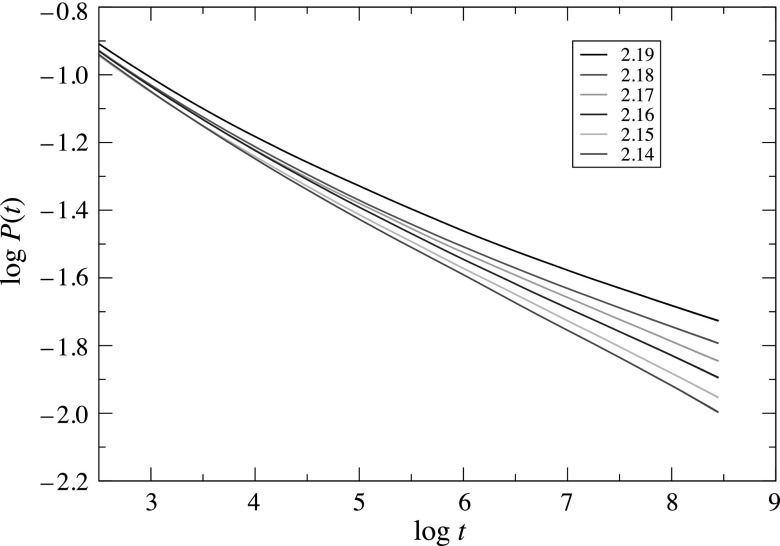
Log-log plot of the survival probability *P*(*t*) versus time for $R_{h}=1.0,\;R_{v}=0.5,\;I_{h}=1.0$, $I_{v}=2.0$, and *D* = 0.5. After an initial transient decay, the curve for $\rho_{v}=2.17$ is the closest to a straight line, indicating the critical point.

In the continuous-time simulations, we choose the vector density *ρ_v_* as the control parameter. With the other parameters fixed at $R_{h}=1.0,\;R_{v}=0.5,\;I_{h}=1.0,I_{v}=2.0,$ and *D* = 0.5, we vary *ρ_v_* to find its critical value. One way of locating the critical point is by the analysis of the curvature of the survival probability, *P*(*t*), a quantity furnished by spreading simulations. As an illustration of the method, we show a log-log plot of *P*(*t*) as a function of *t* in Fig. 1 for *ρ_v_* varying from 2.14 to 2.19. These data were obtained using a system size of *L* = 5000, and a maximum time of $t_{\max}=8000$; the results represent an average over $N_{\text{rep}}=50\;000$ realizations.

As discussed in Sec. III, the critical point is marked by an asymptotic power-law decay of the survival probability. Thus our best estimate for the critical vector density $\rho_{v,c}$ is the value that yields a log-log plot of *P*(*t*) showing the least curvature. In Fig. 1, the curves for $\rho_{v}=2.19$ and 2.18 clearly veer upward, while those for $\rho_{v}=2.14-2.16$ veer downward, giving $\rho_{v,c}=2.17$ as our best estimate. The critical exponent *δ* is (minus) the asymptotic slope of the corresponding curve.

**Fig. 2.**
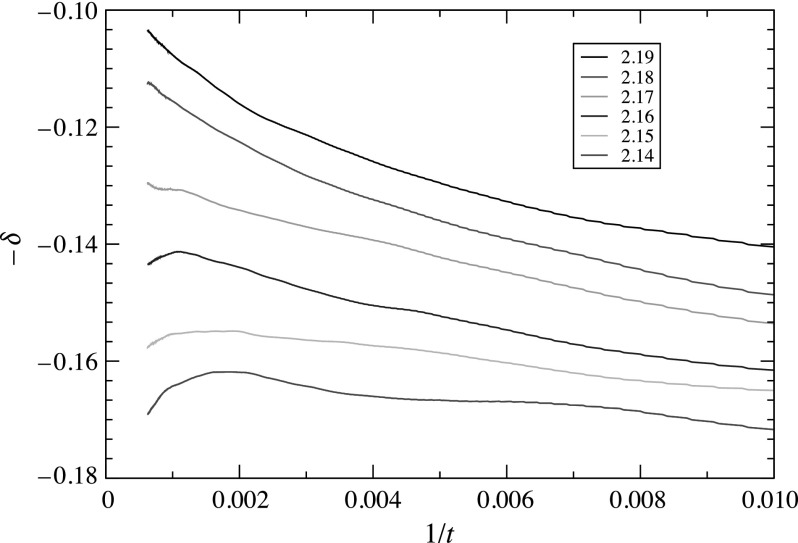
Local slopes −δ(t) for the survival probability versus 1/t for $R_{h}=1.0$, $R_{v}=0.5,\;I_{h}=1.0,\;I_{v}=2.0$, and *D* = 0.5. Notice that the local-slope plot for $\rho_{v}=2.17$ exhibits the least curvature, confirming the value for the critical vector density.

Due to finite-time corrections to scaling, the slope, even for the exact value of $\rho_{v,c}$, exhibits some variation at short times. To estimate the asymptotic slope as t→∞, we compute the slope over blocks of data representing a fixed interval of lnt, and plot the slope versus 1/t. Figure 2 shows that such a local-slope plot is highly sensitive to deviations from criticality, making it easier to decide which curve is nearest criticality. At this level of precision, the critical vector density lies between 2.16 and 2.18, providing $\rho_{v,c}=2.170\;(5)$, where the uncertainty estimate reflects the fact that the values 2.16 and 2.18 are excluded. To improve this estimate, we would have to run the simulation for larger values of *L*, $t_{\max}$, and $N_{\text{rep}}$.

The local-slopes plot allows us to estimate the value of *δ* by extrapolation of the slope to infinite time, yielding δ=0.126 (5).

**Fig. 3.**
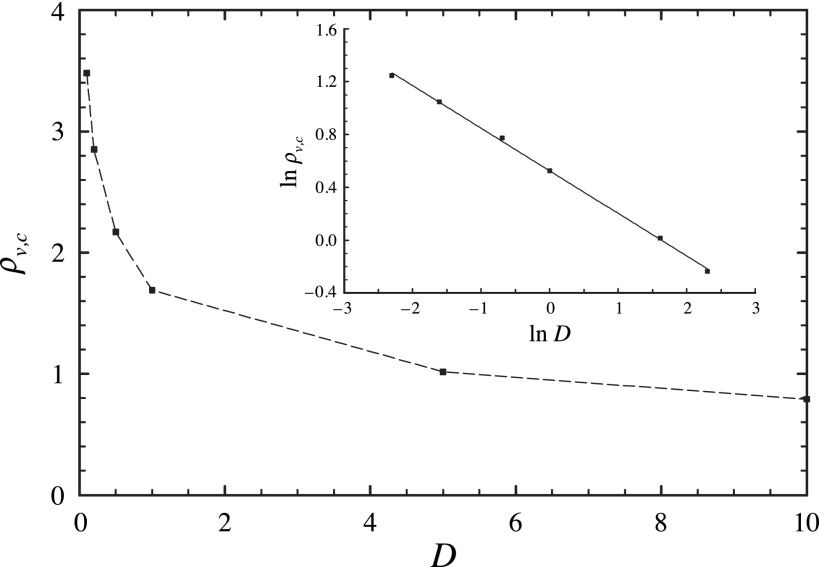
Critical vector density $\rho_{v,c}$ versus the diffusion rate *D*. The other parameters are $R_{h}=1.0,\;R_{v}=0.5,\;I_{h}=1.0$, and $I_{v}=2.0$. Inset: the same data plotted on log scales.

We next consider the critical vector density dependence on the diffusion rate, leaving the other parameters fixed. The results are shown in Fig. 3 from which we can conclude that, as expected, diffusion facilitates the spread of the epidemic, so that the larger the value of *D*, the smaller the threshold density $\rho_{v,c}$. The phase boundary data are well fit by a power law of the form

$$\rho_{v,c}=1.69D^{-0.32},$$ (10)

which relates the critical vector density and the diffusion rate.

We performed complementary discrete-time simulations using the host infection rate *I_h_* as the control parameter. The other parameter values are fixed at $R_{v}=R_{h}=0.5$, $I_{v}=2.0$, *D* = 0.1, and $\rho_{v}=3$. Spreading simulations for a maximum time of 50 000 on lattices of *L* = 4000 sites yield the critical value $I_{h,c}=0.853(3)$.

## VI. CONCLUDING REMARKS

We have discussed methods used to simulate the spread of a vector-borne epidemic in a static population. The algorithms are readily adapted to study other nonequilibrium processes. In particular, we have studied a model of malaria transmission based on Ross' model, but including fluctuations, spatial structure, and diffusion of vectors.^3^ The model is of interest in the context of statistical mechanics because it exhibits a phase transition.

A simple model can be very useful for understanding the principal features of real-world complex systems or processes. We note that the vast majority of models in the epidemiology literature are deterministic and lack spatial structure. Including stochasticity and spatial dependence may lead to improved predictions of epidemic thresholds.

We discussed continuous-time and discrete-time algorithms, providing different approaches to implementing the model. Although the continuous-time algorithm is more faithful to the original Markov process, the discrete time implementation is more efficient computationally. The results of the two approaches agree qualitatively but differ somewhat in details. Because there is usually significant uncertainty regarding the true values of the parameters (transition rates) used in the model, the differences between predictions furnished by the two simulation strategies may not be important. Therefore qualitative results on, say, how to reduce the likelihood of a large epidemic may be of more utility than precise numerical predictions.

Universal properties such as critical exponents have attracted the interest of physicists who study phase transitions but are of limited interest in epidemiology. The simulation methods described here may nevertheless be useful in this broader, more applied context.

We hope that the methods discussed in this paper inspire interested students to consider studying statistical physics or at least appreciate some of the problems currently studied in this area.

## VII. SUGGESTED PROJECTS

(1) Given the set of parameters $R_{h}=1.0,\;R_{v}=0.5,\;I_{h}=1.0,\;I_{v}=2.0$, *D* = 0.5, and $\rho_{v}=2.17$, run the simulation for the one-dimensional vector-borne epidemic model for a lattice size *L* = 5000, a maximum time of $t_{\max}=5000$ and $N_{\text{rep}}=50\;000$ realizations. Compare the values obtained for the survival probability *P*(*t*) at different times with the reference values shown in Table I.
(2) Suppose that due to changes in topography or settlement density, the distance between human host habitations varies in space. We will represent each habitation by a lattice site and represent this situation by a position-dependent diffusion rate, with larger separations corresponding to smaller rates. Adapt the algorithms we have described to the case of a site-dependent diffusion rate *D_i_*. To model heavily and sparsely populated regions, for example, *D_i_* could take two values, generating clumps of sites with the same value. Determine in which regions the epidemic is more likely to persist, and where the density of infected hosts and vectors is greatest.
(3) The algorithms we have described require that a new realization be started each time the absorbing state is reached. A useful alternative, especially in the vicinity of the critical point, is *quasistationary* simulation, in which the absorbing state is effectively removed from the state space. The method is described in detail in Ref. 25. Here, we simply provide a recipe, which runs as follows. We maintain a collection of *N_s_* saved configurations, starting from the initial one (which might have all hosts and vectors infected). In the initial phase of the simulation, we save the current configuration to the list at each time step. Once *N_s_* configurations are saved, we update the list, substituting a configuration on the list with the current configuration, with probability *p_r_* at each unit time interval. (The replacement probability *p_r_* is determined by the condition that the residence time, $N_{s}/p_{r}$ of a configuration on the list be long compared to the mean lifetime of the process, yet short compared to the duration of the simulation.) The evolution of the process proceeds as before, except when an absorbing configuration is reached. In this case, the latter is exchanged for one of the saved configurations, which, by construction, is active. Following an initial transient, this modified process attains the quasistationary distribution; that is, the probability distribution conditioned on not having visited the absorbing state. Averages over this distribution correspond to asymptotic long-time properties of the active process and may be used to infer the critical properties. Use quasistationary simulations to determine the infected host and vector densities as a function of one of the parameters. Convenient values for the quasistationary scheme are $N_{s}=1000$ and $p_{r}=0.001$.

## Figures and Tables

**Table I.** Values obtained for *P*(*t*) at times *t* = 1000, 2000, and 5000.

*t*	*P*(*t*)
1000	0.193(2)
2000	0.176(2)
5000	0.156(2)
